# Transcutaneous vagal nerve stimulation protects against stress‐induced intestinal barrier dysfunction in healthy adults

**DOI:** 10.1111/nmo.14382

**Published:** 2022-04-28

**Authors:** Tamara Mogilevski, Sam Rosella, Qasim Aziz, Peter R. Gibson

**Affiliations:** ^1^ Department of Gastroenterology Monash University and Alfred Health Melbourne Australia; ^2^ Barts Health NHS trust London UK; ^3^ Centre for Neuroscience Surgery and Trauma Blizard Institute Wingate Institute of Neurogastroenterology Barts and the London School of Medicine and Dentistry Queen Mary University of London London UK

**Keywords:** corticotropin releasing hormone (CRH), intestinal barrier dysfunction, intestinal fatty acid binding protein (I‐FABP), transcutaneous vagal nerve stimulation, urine dual sugar test

## Abstract

**Background:**

Intestinal barrier dysfunction is the likely initiating event in multiple human diseases. Currently, there are limited therapeutic strategies to address its dysfunction. Animal studies suggest that vagal nerve stimulation may improve intestinal barrier function, but this has not been evaluated in humans. This study aimed to determine the effect of vagal nerve stimulation on intestinal permeability in adults administered a bolus dose of intravenous corticotropin releasing hormone (CRH) which has been shown to increase small intestinal permeability in healthy human subjects.

**Methods:**

In a cross‐over study, 16 volunteers (median age 34 years, 11 female) were randomized to receive auricular transcutaneous vagal nerve or sham stimulation (10 minutes each side) after intravenous administration of 100 µg of CRH. Intestinal barrier function was measured before and 2 h after each intervention with dual‐sugar urine testing (lactulose:mannitol ratio) and intestinal fatty‐acid binding protein (I‐FABP).

**Key Results:**

Exposure to CRH increased I‐FABP concentrations by a median of 49 (IQR 4‐71)% (*p* = 0.009). Lactulose:mannitol ratios were 0.029 (0.025‐0.050) following vagal stimulation compared with 0.062 (0.032‐0.170) following sham stimulation (*p* = 0.0092), representing a fall of 53 (22‐71)%. I‐FABP concentrations did not change (*p* = 0.90).

**Conclusions:**

Brief non‐invasive vagal nerve stimulation consistently reduces paracellular permeability of the small intestine after CRH administration, but does not entirely mitigate I‐FABP release from the epithelium. Studies of vagal nerve stimulation in disease states are warranted.

## Graphical abstract

A brief period of transcutaneous vagal stimulation reduced paracellular intestinal permeability by 53% compared with sham stimulation after a single dose of corticotropin releasing hormone. These findings warrant further testing in disease states.

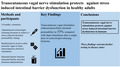

**Key Points**

- Vagal nerve stimulation improves paracellular intestinal permeability.
- Urine lactulose:mannitol ratio and plasma I‐FABP levels represent different aspects of the intestinal barrier function.
- Plasma I‐FABP levels likely represent a degree of trans‐cellular intestinal permeability which is not altered by vagal nerve stimulation.

## 1 INTRODUCTION

The intestinal barrier is a dynamic, semipermeable structure that simultaneously allows absorption of nutrients while protecting the host from potentially harmful effects of the luminal environment. Intestinal permeability is altered by physiological (e.g., stress) and pathological states (e.g., mucosal inflammation). There is accumulating evidence implicating intestinal barrier dysfunction as the initiating event in both gastrointestinal and extra‐gastrointestinal disease states. Despite this, there are currently limited options for its accurate measurement and therapeutic manipulation.

The intestinal barrier can be described as having a trans‐cellular and paracellular route of potential permeability. The urine dual‐sugar test—a paracellular permeability measure—is the current gold standard of non‐invasive intestinal permeability testing.^1^, ^2^ This can, however, be difficult to execute outside of a laboratory environment. Therefore, plasma markers for which change correlates with changes of intestinal permeability have become of interest. The most promising of these is the intestinal fatty acid binding protein (I‐FABP) which is an intracellular protein that is specific to the proximal small intestinal epithelium. It is released into the circulation at times of epithelial injury without cellular disruption.^3^ I‐FABP has been studied, for example, as a potential biomarker to monitor progress in patients with inflammatory bowel disease and coeliac disease.^4^, ^5^, ^6^ Given that I‐FABP is raised at times of intestinal epithelial injury and is itself an intracellular protein, changes in circulating levels of I‐FABP are likely to reflect a degree of modulation of the transcellular route of intestinal permeability, which is not captured by the urine dual‐sugar test.^7^ Furthermore, the correlation of I‐FABP compared with the urine dual‐sugar test has not been previously examined.

Direct electrical stimulation of the vagal nerve seems to play a protective role in modulating intestinal barrier function in animal models with experimental evidence suggesting its ability to reverse stress‐induced changes in intestinal permeability.^8^, ^9^, ^10^ These findings remain to be tested in humans. A non‐invasive mode of vagal nerve stimulation—transcutaneous vagal nerve stimulation—is the optimal tool to study the influence of the vagal nerve on intestinal permeability in human subjects.

The current study aimed to examine the hypotheses that brief vagal stimulation reverses stress‐induced increases in intestinal permeability of healthy human volunteers. The previously published model of intestinal barrier perturbation using corticotropin releasing hormone (CRH) as a model for stress‐induced changes^11^ was utilized. Vagal stimulation was applied using a transcutaneous method and the effects compared with those of sham stimulation. Further, we sought to assess whether changes in I‐FABP concentrations reflected those of the gold‐standard urine dual‐sugar test of intestinal permeability.

## 2 MATERIALS AND METHODS

### 2.1 Participants

Healthy volunteers were recruited from the student and staff community of Queen Mary University of London between March 2019 and June 2019. They were included if they were healthy, aged between 18 and 65 years, and passed an assessment by a gastroenterologist. Exclusion criteria included diabetes, a personal or family history of coeliac disease or inflammatory bowel disease, a personal history of irritable bowel syndrome (IBS) or symptoms consistent with IBS, active ingestion of non‐steroidal anti‐inflammatory medications (with a minimum washout period of 2 weeks after last ingestion), corticosteroids, antibiotics or probiotics, pregnancy or current breastfeeding, known history of anaphylaxis, suspected disorders of the hypothalamic‐pituitary‐adrenal axis, known or suspected cardiac disease or an abnormal screening electrocardiogram and an implanted electrical and/or a neurostimulator device. All participants provided written informed consent.

### 2.2 Protocol

**FIGURE 1:**
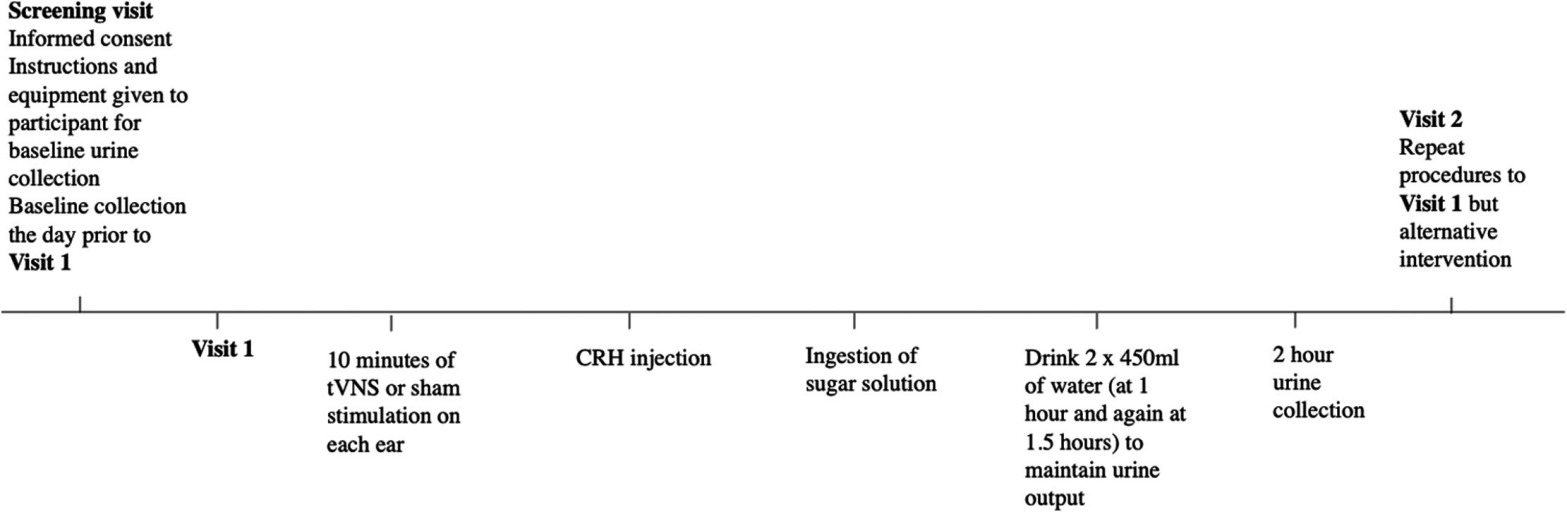
Timeline of interventions during visits. CRH, corticotropin‐releasing hormone

This was a prospective, single‐blinded, placebo‐controlled, crossover study. A timeline of the study procedures is summarized in Figure 1. During a screening visit, participants were assessed by a gastroenterologist (TM) with regard to the inclusion and exclusion criteria, and presence of anxiety or depression. Written informed consent was obtained. Participants were given verbal and written instructions, as well as equipment for their baseline urine collection which they performed at home. This collection formed the baseline urine permeability measurement and was performed by the participants the day prior to visit 1. The subsequent visits were scheduled at a time convenient to the participants. However, the timing of the baseline, visit 1 and visit 2 urine collections were kept constant for each volunteer in order to avoid any diurnal variation effects. Participants arrived for visit 1 after an overnight (or 8 hour) fast. A baseline blood sample was collected. Participants were randomized using the “Randomisation.com” platform to receive either active or sham transcutaneous vagal nerve stimulation. Participants received 10 min of active or sham vagal stimulation to the right followed by 10 min of vagal stimulation to the left ear. They were blinded to the form of intervention they were receiving. Participants then received a 100 μg bolus dose of CRH (Ferring, Kiel, Germany) given slowly intravenously over 30 sec. Blood pressure and heart rate were recorded before and after the injection in order to monitor for adverse events. The participants then emptied their bladder and consumed the sugars for the permeability test. This order of interventions was chosen in order to study the effect of vagal or sham pre‐treatment on intestinal permeability after CRH administration. At 2 h post‐CRH injection, a further blood sample was obtained. Participants then returned 2 weeks after visit 1, where they underwent an identical protocol, but with the alternative intervention. Tolerance for all procedures was assessed by specific questioning. Intolerances or side effects were recorded in the study file of the participants. The protocol was approved by the Queen Mary University of London Ethics Committee (approval number QMERC2018/83) and registered in Clinicaltrials.gov (identifier NCT04061564) as a randomized controlled trial.

### 2.3 Transcutaneous vagal nerve and sham stimulation

Electrical stimulation was delivered transcutaneously using the Digitimer DS7A & DS7AH HV Current Stimulator (Digitimer Ltd, UK) constant current stimulator with an adjusted sine wave current (25 Hz, 200 ms wavelength, 5 V peak to peak amplitude). The current intensity was adjusted by 0.1 mAmp at 5 sec intervals until the maximum current intensity tolerated was achieved. The active form of the intervention (vagal nerve stimulation) was delivered to the concha of the ear and the sham form to the ear lobe using an approved ear clip.^12^ Stimulation was performed for 10 min on the right followed by 10 min on the left ear. Participants were blinded to the type of intervention they were receiving. The up‐titration of current was performed in the same manner for both the active and sham versions of the intervention.

### 2.4 Dual‐sugar intestinal permeability testing

The dual‐sugar permeability test was performed in accordance to previously published literature aiming to assess small intestinal permeability.^13^ Briefly, participants abstained from heavy exercise (70% or more of maximum intensity), alcohol ingestion, and smoking for 3 days prior. After an overnight fast, they voided any remaining urine in their bladder prior to drinking a solution containing 5 g lactulose and 2 g mannitol, diluted in 450 ml of filtered water. Their urine was collected for 2 hours with two 500 ml bottles of water consumed at 1 h and 1.5 h after initiation of the study in order to increase urine output over this period. Participants remained in the laboratory while watching a light‐hearted video of their choice during the 2 h period of urine collection. The final volume of urine was recorded, and the urine was centrifuged (to remove gross debris) prior to storage in aliquots at −80°C. All urine collection bottles contained 1 ml 20% chlorhexidine (Merk, Dorset, United Kingdom) in order to prevent bacterial contamination.

On de‐frosting, 500 µl urine was treated with 1ml acetonitrile, mixed for 30 sec, and then, centrifuged at 12500 rpm and 4°C for 10 min. The supernatant was transferred to a clean tube and dried down at 55°C under nitrogen. The precipitate was reconstituted in 500 µl water and transferred to a high‐performance liquid chromatography (HPLC) vial. The urine samples were run against a calibration curve 15–3000 µg ml^−1^, prepared in a matching matrix. Analysis was carried out using a Nexera HPLC system (Shimadzu), fitted with a Refractive Index Detector (RID), on a Rezex RCM Monosaccharide Ca^2^ ^+^ column (300 × 7.8 mm, Phenomenex) with water as the mobile phase. The flow rate was 0.6 ml min^−1^. The column oven was set to 85°C and the RID cell was set to 60°C. The run time was 30 min. The limits of detection for lactulose samples were between 4.5 and 2987.3 µg/ml, whereas those for mannitol were 16.9 and 30004.0 µg/ml. Lactulose:mannitol ratio was calculated by dividing the lactulose concentration by the paired mannitol concentration and the fractional excretion of the sugars were calculated using previously published methodology.^14^

### 2.5 Blood evaluation

Peripheral venous blood samples were collected into EDTA tubes. They were placed on ice immediately and centrifuged (4°C, 15 min, 1500 × *g*) within 20 min of collection. Plasma was stored in aliquots at −80°C until assayed. Concentrations of I‐FABP were measured in freshly‐thawed plasma by ELISA (R&D Systems, Minneapolis, USA), according to the manufacturers’ protocols. Coefficient of variation between duplicate samples was below 10%. Averages of duplicates were determined, and absolute values were expressed in ng ml^−1^.

### 2.6 Statistical evaluation

Descriptive statistics are presented as median with 1^st^ and 3^rd^ interquartile ratios (IQR), unless otherwise stated. The differences between the endpoints under different conditions were compared using the Wilcoxon matched pairs signed rank test and order of intervention analyses using the Mann–Whitney‐*U* test. The level of statistical significance was set at *p* ≤ 0.05. Correlation was assessed using the Spearman correlation coefficient. All statistical analyses were performed using Prism V8.30 (GraphPad Software LLC).

## 3 RESULTS

### 3.1 Participants

16 healthy volunteers were recruited with a median age of 34 (range 20–62) years. 5 (69%) were female. The median body mass index was 22.1 (IQR 21.9–23.5) kg m^−2^. Only one participant was actively taking non‐steroidal anti‐inflammatory medications requiring a 2 week washout period prior to proceeding with the study. No participant suffered from anxiety or depression within this cohort. All interventions (exposure to CRH, vagal and sham stimulation) were very well tolerated with no reported adverse events. 10 of the 16 participants experienced a mild flushing episode after the CRH injection. 2 of the 16 participants reported a nervous feeling and 1 of the 16 participants reported a metallic taste in their mouth. All side effects were mild and their duration was less than 5 min.

### 3.2 The effect of CRH on intestinal permeability

**FIGURE 2:**
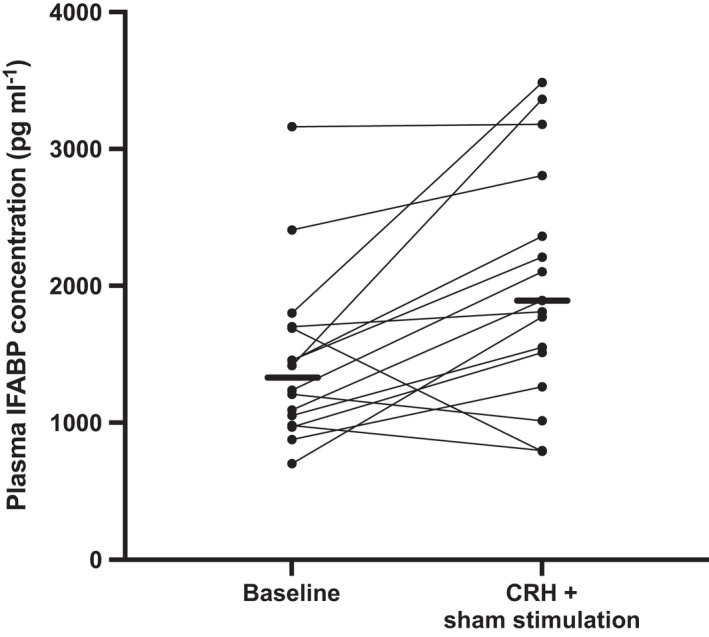
Comparison of plasma concentrations of intestinal fatty acid binding protein (I‐FABP) during the baseline period with those after corticotropin‐releasing hormone (CRH) exposure 2 h after 20 min sham stimulation. Concentrations of I‐FABP increased with CRH exposure (*p* = 0.009; Wilcoxon signed‐rank test). The horizontal bars represent the median values

Concentrations of I‐FABP increased by a median of 521 pg ml^−1^ after CRH, representing a change relative to baseline of 49 (4–71)% (*p* = 0.009) (Figure 2). This confirmed small intestinal epithelial injury in response to parenteral injection of CRH. The results of the baseline measurements of the lactulose:mannitol ratio were highly variable with 8 of 16 results being well above the normal range under resting conditions for our testing (Figure S1B). They did not change overall after CRH exposure. For those who did have lactulose:mannitol ratios within acceptable limits (defined for the purposes of this study as <0.035 based on previous data).^11^, ^15^ CRH exposure did induce a significant increase in the lactulose:mannitol ratio (Figure S1A). Adherence to the duration and timing of home collection of urine and other factors such as resting, avoidance of stress, and food restrictions was considered poor in many.

### 3.3 The effect of transcutaneous vagal stimulation on intestinal permeability

**FIGURE 3:**
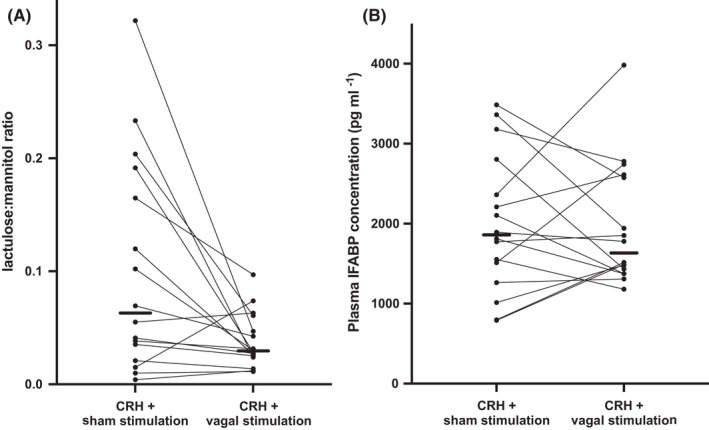
Comparison of barrier measures after corticotropin‐releasing hormone (CRH) stimulation followed by sham vs transcutaneous vagal stimulation. A. Lactulose:mannitol ratios: vagal stimulation was associated with a reduced lactulose:mannitol ratio compared with those associated with sham stimulation (*p* = 0.009; Wilcoxon matched‐pairs signed rank test). B. Plasma I‐FABP concentrations: there was no significant difference between sham and vagal stimulation (*p* = 0.90). The horizontal bars represent median values

The effect of vagal nerve stimulation was determined by the paired comparison with biomarkers after sham stimulation and the results are shown in Figure 3. The lactulose:mannitol ratio was 0.062 (0.032–0.170) following sham stimulation, which was greater than 0.029 (0.025–0.050) (*p* = 0.0092) following vagal nerve stimulation. This represented a difference of 53 (22–71)%. There was a significant reduction of fractional lactulose excretion in the vagal stimulation compared with the sham stimulation treatments; 0.1 (0.7–0.13)% vs 0.26 (0.09–0.6)% (*p* = 0.03) and no difference in the fractional excretion of mannitol [(0.085 (0.06–0.12)% sham vs 0.097 (0.06–0.11)% with vagal stimulation; *p* = 0.98].

In contrast, I‐FABP concentrations were not different after sham stimulation compared with those after vagal nerve stimulation (*p* = 0.90, Figure 3). The relative change from baseline was blunted by vagal stimulation with an increase of 25 (−14–60)% (*p* = 0.19) compared the statistically significant increase of median 49% (*p* = 0.009) with sham stimulation, as shown in Figure 2.

**FIGURE 4:**
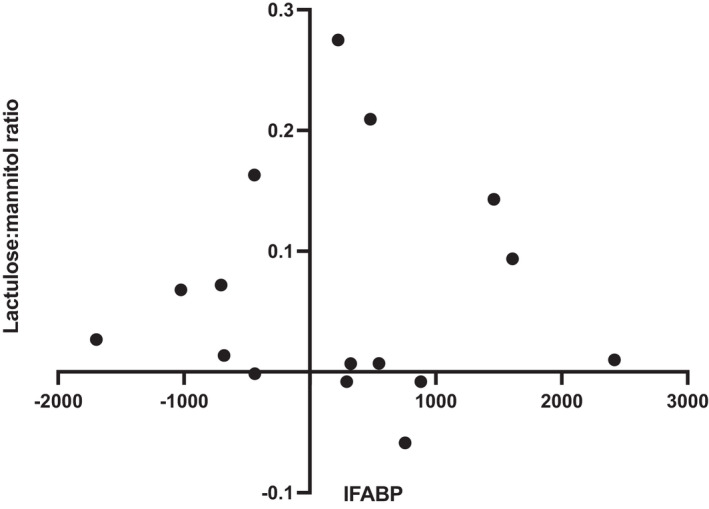
Comparison of the change in lactulose:mannitol ratio with plasma concentrations of intestinal fatty acid binding protein (I‐FABP) in sham versus vagal stimulation following corticotropin‐releasing hormone (CRH; Spearman's *r* = −0.16; *p* = 0.55)

8 participants received the sham stimulation intervention as their first intervention and 8 received vagal stimulation, with no order effects observed. Likewise, there were no differences between the I‐FABP values at any of the time points when comparing the order of intervention received. Further, there was no order effect detected in the baseline I‐FABP levels. The difference between lactulose:mannitol ratio and plasma I‐FABP in the vagal versus sham interventions levels did not correlate (Spearman's *r* = −0.16; *p* = 0.56; Figure 4).

## 4 DISCUSSION

The changes in intestinal barrier function are of pathogenic importance in several gut disorders. This unifying abnormality underpins a group of conditions that range widely from Crohn's disease to irritable bowel syndrome, food allergies, and hepatic encephalopathy; thereby posing a significant clinical burden. Apart from powerful anti‐inflammatory therapies in patients with gross intestinal inflammation,^16^ there is a general lack of therapeutic or preventive approaches to specifically modulate barrier function and the “leaky bowel.” The current study has shown that short‐duration transcutaneous vagal nerve stimulation in healthy adults can reduce paracellular permeability associated with a stressful stimulus, but not the release of I‐FABP from small intestinal epithelium.

There is abundant animal and human evidence to suggest that peripheral administration of CRH induces paracellular and trans‐cellular intestinal barrier dysfunction when assessed via both in vivo and invasive modes of intestinal permeability measurements. Changes in both tight junction modulation as well as alteration of trans‐cellular transportation have been documented. ^17^, ^18^, ^19^, ^20^, ^21^, ^22^ This is thought to occur via direct action of CRH on CRH‐specific receptors on enterocytes, mast cells, eosinophils, and mononuclear cells, as reviewed elsewhere.^23^, ^24^, ^25^ The present study confirms the aforementioned findings by documenting, for the first time, a significant rise in I‐FABP after CRH administration. These findings are concordant with other in vivo and invasive modes of permeability measurements. A lack of adherence to the protocol during the unsupervised, at‐home baseline dual‐sugar urine test measurements led to higher‐than‐expected lactulose:mannitol ratios, some being implausibly high. Those with normal lactulose:mannitol ratios did show increased lactulose:mannitol ratios after CRH exposure. However, the data were considered too unreliable to include in the main results. This lack of adherence highlights the limitations of this method of intestinal permeability measurement in the clinical setting and supports the use of close supervision in a calm environment during the period of urine collection.

Transcutaneous stimulation of the vagus was associated with a halving of paracellular intestinal permeability after CRH‐induced stress compared with those following sham stimulation. This interpretation is predicated on three assumptions: first, that the changes in lactulose:mannitol ratio are not due to the alterations in gut motility by CRH and vagal stimulation, respectively; secondly, that vagal nerve stimulation by the transcutaneous method was successful; and lastly, that sham stimulation was indeed sham. There is evidence to suggest that vagal nerve stimulation, and to a lesser extent CRH, increases gastroduodenal motility^26^, ^27^ and a theory to suggest that accelerated intestinal transit may decrease contact time between sugar probes and the intestinal epithelium, thus impairing absorption. This has not borne out in previous intestinal permeability studies.^14^, ^28^ Furthermore, the potential for this effect is mitigated by using the ratio of two sugar probes. Therefore, intestinal transit alterations from CRH and vagal stimulation are unlikely to have had an impact on our findings. The last two assumptions are backed by previous experience with transcutaneous vagal nerve stimulation using the same stimulation mechanism in esophageal hypersensitivity, where it was shown that pre‐treatment with vagal stimulation reduced acid‐induced esophageal hypersensitivity in a healthy volunteer model.^29^ Further, functional magnetic resonance imaging studies reveal that the transcutaneous mode of vagal stimulation bears a similar degree of vagal fiber activation to that of invasive vagal stimulation, whereas sham stimulation does not.^30^, ^31^, ^32^

Despite the consistently lower lactulose:manitol ratio following vagal vs sham stimulation, plasma concentrations of I‐FABP were not different, although there was a signal that vagal stimulation had some impact on I‐FABP release. The consistent increases in I‐FABP levels in the sham arm were not observed with vagal stimulation, which could arguably be described as a blunting of the response or partial compensation for the injury associated with CRH stimulation. The differences in response to the markers are likely to reflect the different pathways of intestinal permeability that these two tests represent.

Our findings of improvement in the intestinal barrier post transcutaneous vagal stimulation mimic those described in mice models using invasive vagal nerve stimulation.^33^, ^34^ These murine studies give mechanistic insights. At the cellular junction level, pre‐treatment with vagal nerve stimulation decreased the expression of myosin light chain kinase and mucosal tumor necrosis factor‐alpha, while increasing expression of occludin,^33^ thereby tightening the paracellular pathway of intestinal permeability. These mechanisms may explain findings in the present study, where vagal stimulation had a greater effect when measured by the urine dual‐sugar test (a measure of paracellular intestinal permeability) as compared with I‐FABP which is released from viable but injured epithelial cells and would more likely reflect some changes in transcellular protein kinetics.^7^ These effects of vagal nerve stimulation are likely to be communicated to the intestinal mucosa via glial cells which are activated in response to the treatment and modulation of dendritic cells in the mesenteric lymph.^8^, ^34^ This is thought to occur by local activation of the α‐7‐nicotinic‐acetylcholine receptor in the enteric nervous system.^21^

Together with anti‐inflammatory effects^23^ and the reduction of visceral sensitivity demonstrated in the human esophagus,^29^ such protective effects of transcutaneous vagal stimulation signal its potential application in several chronic human conditions associated with inflammation, visceral hypersensitivity, and increased intestinal permeability such as inflammatory bowel disease and irritable bowel syndrome. Indeed, there has been recent interest in using vagal nerve stimulation in patients with Crohn's disease^35^, ^36^ and in extra‐gastrointestinal inflammatory conditions.^23^

The strengths of this study were its utilization of a multimodal approach to intestinal barrier measurement. The main limitation in this exploratory study was a lack of an objective marker to indicate the success of vagal nerve stimulation. However, the difference between the post‐intervention lactulose:mannitol ratios do highlight a mechanism of action of vagal nerve stimulation at the local intestinal level not previously explored in humans.

In conclusion, as little as 20 min of transcutaneous vagal stimulation normalizes paracellular intestinal permeability, reducing it by over 50% compared with sham in healthy subjects after a single dose of CRH. These results highlight the importance of examining different pathways of the intestinal barrier when designing future studies. Transcutaneous vagal nerve stimulation, as a non‐pharmacological, low‐cost intervention with minimal side effects, warrants testing in clinical settings to determine its therapeutic effect.

## AUTHOR CONTRIBUTIONS

TM: Study concept and design, acquisition of data, analysis and interpretation of data, drafting of the manuscript; critical revision of the manuscript for important intellectual content, statistical analysis, obtained funding. SR: Laboratory support and completion of ELISA protocols, critical revision of the manuscript. QA: Study concept and design, drafting of the manuscript, critical revision of the manuscript for important intellectual content, help with obtaining funding. PRG: Study concept and design, analysis and interpretation of data, drafting of the manuscript, critical revision of the manuscript for important intellectual content, statistical analysis.

## CONFLICT OF INTEREST

PRG has served as a consultant or advisory board member for Anatara, Atmo Biosciences, Falk Pharma, Immunic Therapeutics, Novozymes, Novoviah, Comvita and Takeda. He has received research grants for investigator‐driven studies from Atmo Biosciences. He holds shares in Atmo Biosciences. His department financially benefits from the sales of a digital application, booklets, and online courses on the FODMAP diet. QA holds grant for a commercial trial from Classado and is a director of “My Health Chart.” TM and SR have no conflicts of interest to declare.

## Supporting information

Fig S1
