# Supplementary material for: Transcutaneous vagal nerve stimulation protects against stress‐induced intestinal barrier dysfunction in healthy adults
Source: Neurogastroenterol Motil. 2022 Apr 28;34(10):e14382. doi: 10.1111/nmo.14382 (PMC9786250; doi:10.1111/nmo.14382)
Supplement: Supplementary file 1 — Fig S1 [file NMO-34-e14382-s001.docx]

**Supplementary figure 1:**

Comparison of baseline lactulose:mannitol to post sham stimulation and CRH intervention.

The median baseline of 0.038 (range 0.009-0.59) was higher than expected in this cohort of healthy volunteers due to an overall poor adherence to the protocol. It was expected that the healthy participants’ lactulose:mannitol ratios would be below 0.035. The fractional excretion of lactulose at baseline was 0.12% (0.1 - 0.15) and mannitol 9.1% (5.4 - 10.3) – figures 1A and 1B.

Figure 1C represents the 8 patients in whom the baseline lactulose:mannitol ratio was below 0.035 and Figure 1D represents participants in whom the baseline lactulose:mannitol ratio was above 0.035. Bars represent the medians.

In the group of participants with lower baseline lactulose:mannitol ratios; parenteral injection of CRH increased the lactulose:mannitol ratio from median 0.023 to 0.11 (0.021 – 0.22); *p = 0.039 (Wilcoxon signed rank test). There was no difference between the baseline and post intervention lactulose:mannitol ratios in the group of participants with higher-than-expected baseline lactulose:mannitol ratios (0.057 to 0.055; p = 0.25).

**1A 1B**

**1C 1D**
